# Correction: Cu(i) diimine complexes as immobilised antibacterial photosensitisers operating in water under visible light

**DOI:** 10.1039/d4ma90119c

**Published:** 2024-11-11

**Authors:** Martin V. Appleby, Peter G. Walker, Dylan Pritchard, Sandra van Meurs, Carly M. Booth, Craig Robertson, Michael D. Ward, David J. Kelly, Julia A. Weinstein

**Affiliations:** a Department of Chemistry, University of Sheffield Sheffield S3 7HF UK Julia.Weinstein@sheffield.ac.uk; b Department of Molecular Biology and Biotechnology, University of Sheffield UK D.Kelly@sheffield.ac.uk; c Department of Chemistry, University of Warwick Coventry CV4 7AL UK

## Abstract

Correction for ‘Cu(i) diimine complexes as immobilised antibacterial photosensitisers operating in water under visible light’ by Martin V. Appleby *et al.*, *Mater. Adv.*, 2020, **1**, 3417–3427, https://doi.org/10.1039/D0MA00642D.

The authors regret that a chemical structure was depicted incorrectly in Fig. 1 and Scheme S1. The corrected figures are as shown below.

**Fig. 1:**
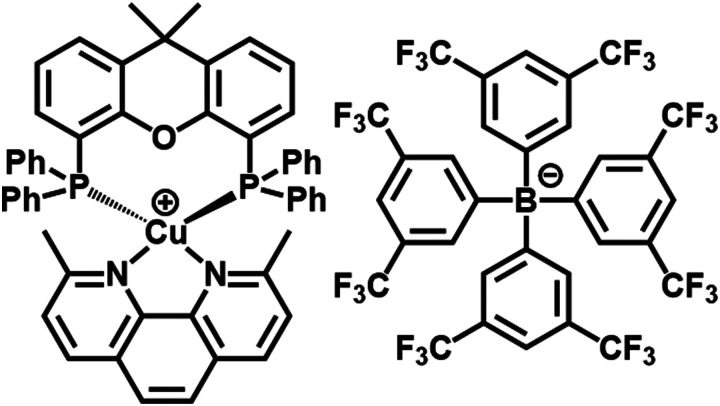
Chemical structure of complex **1** with counterion tfpb^−^. Structure of complex **1** obtained by single crystal X-ray crystallography, which is fully consistent with that published previously, is given in the ESI,† Fig. S8. CCDC 2012235.


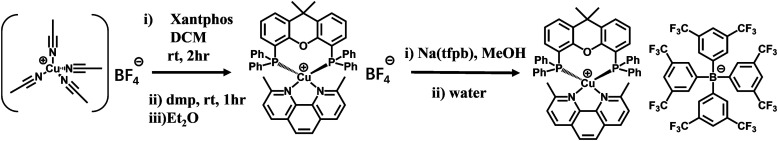
 **Scheme S1** Synthesis scheme of complex **1**.^1^

The Royal Society of Chemistry apologises for these errors and any consequent inconvenience to authors and readers.

